# Publisher Correction: An achromatic metafiber for focusing and imaging across the entire telecommunication range

**DOI:** 10.1038/s41467-022-32153-y

**Published:** 2022-07-26

**Authors:** Haoran Ren, Jaehyuck Jang, Chenhao Li, Andreas Aigner, Malte Plidschun, Jisoo Kim, Junsuk Rho, Markus A. Schmidt, Stefan A. Maier

**Affiliations:** 1 School of Physics and Astronomy, Faculty of Science, Monash University, Melbourne, Victoria 3800 Australia; 2 Chair in Hybrid Nanosystems, Nanoinstitute Munich, Faculty of Physics, Ludwig Maximilian University of Munich, Munich, 80539 Germany; 3 Department of Chemical Engineering, Pohang University of Science and Technology (POSTECH), Pohang, 37673 Republic of Korea; 4 Leibniz Institute of Photonic Technology, 07745 Jena, Germany; 5 Abbe Center of Photonics and Faculty of Physics, FSU Jena, 07745 Jena, Germany; 6 Department of Mechanical Engineering, Pohang University of Science and Technology (POSTECH), Pohang, 37673 Republic of Korea; 7 POSCO-POSTECH-RIST Convergence Research Center for Flat Optics and Metaphotonics, Pohang, 37673 Republic of Korea; 8 Otto Schott Institute of Material Research, FSU Jena, 07745 Jena, Germany; 9 Department of Physics, Imperial College London, London, SW7 2AZ UK

**Keywords:** Metamaterials, Imaging and sensing, Laser material processing, Applied optics

Correction to: *Nature Communications* 10.1038/s41467-022-31902-3, Article published online 19 July 2022

In this article the affiliation details for Chenhao Li were incorrectly given as ‘School of Physics and Astronomy, Faculty of Science, Monash University, Melbourne, Victoria 3800, Australia’ but should have been ‘Chair in Hybrid Nanosystems, Nanoinstitute Munich, Faculty of Physics, Ludwig Maximilian University of Munich, Munich 80539, Germany’. The original article has been corrected.

In this article the affiliation details for Andreas Aigner were incorrectly given as ‘School of Physics and Astronomy, Faculty of Science, Monash University, Melbourne, Victoria 3800, Australia’ but should have been ‘Chair in Hybrid Nanosystems, Nanoinstitute Munich, Faculty of Physics, Ludwig Maximilian University of Munich, Munich 80539, Germany’. The original article has been corrected.

