# Supplementary material for: An achromatic metafiber for focusing and imaging across the entire telecommunication range
Source: Nat Commun. 2022 Jul 19;13:4183. doi: 10.1038/s41467-022-31902-3 (PMC9296535; doi:10.1038/s41467-022-31902-3)
Supplement: Supplementary file 1 — Supplementary Information [file 41467_2022_31902_MOESM1_ESM.pdf]

Supplementary Information for

An achromatic metafiber for focusing and imaging across the entire telecommunication range

Haoran Ren[1, 2, *, †], Jaehyuck Jang[3, †], Chenhao Li[1, †], Andreas Aigner[1], Malte Plidschun[4, 5],

Jisoo Kim[4, 5], Junsuk Rho[3, 6, 7, *], Markus A. Schmidt[4, 5, 8, *]and Stefan A. Maier[1, 2, 9, *]

[1] Chair in Hybrid Nanosystems, Nanoinstitute Munich, Faculty of Physics, Ludwig-Maximilians-Universität München, München, 80539, Germany.

[2] School of Physics and Astronomy, Faculty of Science, Monash University, Clayton, Victoria 3800, Australia.

[3] Department of Chemical Engineering, Pohang University of Science and Technology (POSTECH), Pohang, 37673, Republic of Korea.

[4] Leibniz Institute of Photonic Technology, 07745 Jena, Germany.

[5] Abbe Center of Photonics and Faculty of Physics, FSU Jena, 07745 Jena, Germany.

[6] Department of Mechanical Engineering, Pohang University of Science and Technology (POSTECH), Pohang, 37673, Republic of Korea.

[7] POSCO-POSTECH-RIST Convergence Research Center for Flat Optics and Metaphotonics, Pohang 37673, Republic of Korea

[8] Otto Schott Institute of Material Research, FSU Jena, 07745 Jena, Germany.

[9] Department of Physics, Imperial College London, London SW7 2AZ, United Kingdom.

*Emails: Haoran.Ren@monash.edu; jsrho@postech.ac.kr; Markus.Schmidt@leibniz-ipht.de ; Stefan.Maier@monash.edu.

† These authors contributed equally to this work.

Inventory of Supplementary Information file:

Supplementary Note 1. Group delay modulation based on a 3D meta-atom.

Supplementary Note 2. Derivation of the time-bandwidth product of a nanopillar waveguide.

Supplementary Note 3. Spherical lens and group delay functions.

Supplementary Note 4. Fiber wavefront correction.

Supplementary Note 5. Time-bandwidth product of a waveguide-type achromatic metalens.

Supplementary Note 6. Polarization-insensitive 3D meta-atom design based on cross polarization.

Supplementary Note 7. Meta-atom size and pitch considerations.

Supplementary Note 8. Characterization of off-axis focusing of an achromatic metalens.

Supplementary Note 9. Laser damage threshold of a polymer metalens.

Supplementary Figures 1-19.

Supplementary Tables 1-6.

Supplementary References.

Supplementary Note 1. Group delay modulation based on a 3D meta-atom

A 3D nanopillar meta-atom (Fig. S2A) can be considered as an individual waveguide, with effective refractive indices of n_{TE} and n_{TM} for incident light polarized along the long and short axis, respectively (Fig. S2B-2D). The propagation phase for the polarization along the long axis (Φ_{TE}) and the short axis (Φ_{TM}) can be written as:

$$\phi_{TE,TM}(\omega, H) = \frac{\omega}{c} n_{TE,TM}(\omega) H \tag{1}$$

where c is the speed of light. The group delay $\frac{d\phi_{TE,TM}}{d\omega}$, which is the first order derivative of the phase response with respect to the angular frequency, which can be calculated as:

$$\frac{d\phi_{TE,TM}}{d\omega} = \frac{1}{c}\left(n_{TE,TM}(\omega) + \omega \frac{dn_{TE,TM}(\omega)}{d\omega}\right) H. \tag{2}$$

Unlike conventional planar metasurfaces consisting of 2D meta-atoms with a fixed height, the height degree of freedom of our 3D meta-atoms can largely modulate the group delay response and reach a wide tuning range (Fig. S2E-2G).

Supplementary Note 2. Derivation of the time-bandwidth product of a nanopillar waveguide.

For the light propagation in a waveguide with restricted transverse extension, the phase constant of the waveguide equals the wavenumber in the vacuum (k_0) times the effective refractive index (n_{eff}): $k = n_{\text{eff}} k_0 = n_{\text{eff}} \frac{\omega}{c}$, where ω is the angular frequency and c is the speed of light. The nanopillar waveguide can be regarded as a dielectric lossless slow-light device, its group velocity v_g can be derived as:

$$v_g = \frac{\partial\omega}{\partial k} = \frac{c}{n_{\text{eff}}+\omega\frac{dn_{\text{eff}}}{d\omega}}. \tag{3}$$

The group index $S(\omega)$ can be defined as:

$$S(\omega) = \frac{c}{v_g} = n_{eff} + \omega\frac{dn_{eff}}{d\omega}. \tag{4}$$

For a polymer nanopillar waveguide used in our interested spectral range, the dispersion of the effective refractive index is linear to the frequency (Fig. S2), Eq. 4 can be described as:

$$\frac{\text{d}n_{\text{eff}}}{d\omega} = \frac{n_{\text{max}}-n_{\text{min}}}{\omega_{\text{max}}-\omega_{\text{min}}}, \tag{5}$$

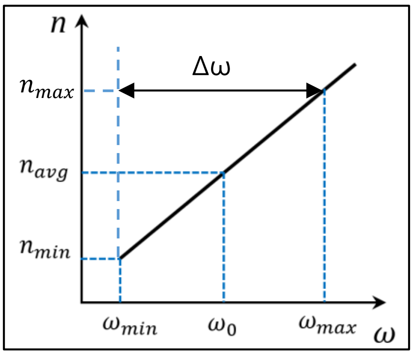

where n_{min} and n_{max} are the minimum and maximum effective refractive indices of the waveguide across the frequency range from ω_{min} to ω_{max}. Therefore, the group index at a central frequency (ω_0) can be rewritten as:

$$S(\omega_0) = n_{eff}(\omega_0) + \frac{\omega_0(n_{max}-n_{min})}{\Delta\omega}. \tag{6}$$

The group velocity of the ideal device can therefore be calculated as:

$$v_g = \frac{c}{S(\omega_0)} = \frac{c}{n_{eff}(\omega_0)+\frac{\omega_0(n_{max}-n_{min})}{\Delta\omega}}. \tag{7}$$

The time delay of the waveguide medium T with height H can be derived as:

$$T = H\left(\frac{1}{v_g} - \frac{1}{c}\right) = H\left(\frac{n_{eff}(\omega_0) - 1 + \frac{\omega_0(n_{max} - n_{min})}{\Delta\omega}}{c}\right) = H\left(\frac{n_{eff}(\omega_0) - 1}{c} + \frac{\omega_0(n_{max} - n_{min})}{c\Delta\omega}\right). \quad (8)$$

Therefore, the time-bandwidth product becomes

$$T\Delta\omega = H\left[\frac{n_{eff}(\omega_0) - 1}{c}(\omega_{max} - \omega_{min}) + \frac{(n_{max} - n_{min})}{c}\omega_0\right]. \quad (9)$$

In general, the frequency bandwidth of a slow-light waveguide is much smaller than the central frequency: $\omega_{max} - \omega_{min} \leq \omega_0$, Eq. R8 can be further reduced to:

$$T\Delta\omega = \frac{\omega_0}{c}H(n_{max} - n_{min}), \quad (10)$$

which is consistent with the Eq. 1 result in the main text. Therefore, the time-bandwidth product in Eq. 10 (same as Eq. 1 in the main text) is mainly contributed from the dispersion of the effective refractive index of an individual nanopillar waveguide.

Supplementary Note 3. Spherical lens and group delay functions

The hyperbolic phase profile of a spherical lens is given as:

$$\varphi(r, \omega, F) = -\frac{\omega}{c}\left(\sqrt{r^2 + F^2} - F\right) \quad (11)$$

where $F = f * \omega^n$ (n = 0 for an achromatic lens); *r*: radial coordinate; *ω*: target angular frequency; *c*: speed of light; *f*: focal length. To explore the dispersion of the lens function, Taylor expansion of $\varphi(r, \omega, F)$ at the minimum angular frequency ω_{min} is implemented as:

$$\varphi(r, \omega, F) = \varphi(r, \omega_{min}, F) + \left.\frac{d\varphi}{d\omega}\right|_{\omega=\omega_{min}}(\omega - \omega_{min}) + \left.\frac{d^2\varphi}{d\omega^2}\right|_{\omega=\omega_{min}}(\omega - \omega_{min})^2 + \cdots$$

(12)

Suppose high-order derivatives (from the second-order expansion term) can be neglected, and F=*f* is invariant with respect to wavelength for an achromatic lens, we

have:

$$\varphi(r,\omega) = -\frac{\omega_{min}}{c}\left(\sqrt{r^2+f^2}-f\right) + \left.\frac{d\varphi}{d\omega}\right|_{\omega=\omega_{min}}(\omega-\omega_{min})$$

$$= -\frac{\omega_{min}}{c}\left(\sqrt{r^2+f^2}-f\right) - \frac{1}{c}\left(\sqrt{r^2+f^2}-f\right)(\omega-\omega_{min}). \quad (13)$$

The derivative of phase $\varphi(r,\omega)$ with respect to ω results in the group delay $\frac{d\varphi(r,\omega)}{d\omega}$, which becomes zero at $r = 0$. To avoid the zero-group delay at the center of the lens, a linear phase shift $\frac{\delta}{\omega_{max}-\omega_{min}}(\omega-\omega_{min})$ is further added to the group delay equation[1]:

$$\tilde{\varphi}(r,\omega) = -\frac{\omega_{min}}{c}\left(\sqrt{r^2+f^2}-f\right) - \frac{1}{c}\left(\sqrt{r^2+f^2}-f\right)(\omega-\omega_{min})$$

$$+\frac{\delta}{\omega_{max}-\omega_{min}}(\omega-\omega_{min})$$

$$= -\frac{\omega_{min}}{c}\left(\sqrt{r^2+f^2}-f\right) - \left(\frac{\sqrt{r^2+f^2}-f}{c} - \frac{\delta}{\omega_{max}-\omega_{min}}\right)(\omega-\omega_{min}). \quad (14)$$

where δ is the maximum phase compensation from ω_{min} to ω_{max} based on the meta-atom design space. The modified group delay $\frac{d\tilde{\varphi}}{d\omega}$ is therefore given as:

$$\frac{d\tilde{\varphi}(r,\omega)}{d\omega} = -\left(\frac{\sqrt{r^2+f^2}-f}{c} - \frac{\delta}{\omega_{max}-\omega_{min}}\right). \quad (15)$$

To achieve the design of an achromatic metalens in an interested frequency range (ω_{min}, ω_{max}), meta-atoms need to be selected to satisfy both the phase $\varphi(r,\omega)$ and the modified group delay $\frac{d\tilde{\varphi}(r,\omega)}{d\omega}$ at the radial coordinate r.

Supplementary Note 4. Fiber wavefront correction

The divergent wavefront of a single-mode fiber (SMF) output can be considered as a Gaussian beam with an initial beam waist w_0, and this beam propagates along the axial direction (perpendicular to the fiber end face) through a surrounding medium with refractive index n_m. The confocal parameter (or Rayleigh range) z_0 is given as[2]:

$$z_0 = \frac{w_0^2}{M^2} * \frac{\pi * n_m}{\lambda}, \tag{16}$$

where the beam quality factor in the medium is presented as:

$$M^2 = \frac{\pi n_m w_0}{\lambda} * asin\left(\frac{NA_{fiber}(\lambda)}{n_m}\right), \tag{17}$$

where λ is the free-space wavelength, and $NA_{fiber}(\lambda)$ is the numerical aperture of the fiber at the wavelength λ. Therefore, the beam waist of the fiber output beam at a distance z is given as:

$$w(z) = w_0\sqrt{1 + \left(\frac{z}{z_0}\right)^2}. \tag{18}$$

Here we set the maximum waist to be $w(z_{prop})$ = 50 μm (a mode field diameter of $2w$=100 μm), resulting in the height of a supporting tower structure on top of the SMF end face to be z_{prop} = 525 μm. As such, the SMF fiber output beam travels a distance of z_{prop} until it reaches our designed achromatic metalens. According to the Fresnel diffraction, the associated phase wavefront $\varphi_{div}(r,\omega,z)$ at z_{prop} is given as:

$$\varphi_{div}(r,\omega,z_{prop}) \approx \frac{\omega}{c} z\left(1 + \frac{r^2}{2({z_{prop}}^2 + z_0^2)}\right) = \frac{\omega}{c} z\left(1 + \frac{r^2}{2z_{prop}R(z_{prop})}\right). \tag{19}$$

Since the propagation distance $z_{prop} \gg z_0$ and $R(z) \approx z$, Eq. 19 can be approximated as:

$$\varphi_{div}(r,\omega,z_{prop}) \approx \frac{\omega}{c}\left(\frac{2{z_{prop}}^2+r^2}{2z_{prop}}\right). \tag{20}$$

Based on the parabolic approximation[3], the fiber acts as a defocusing lens with a focal length of z_{prop}, leading to the overall required phase profile of an achromatic metalens on fiber (Fig. S5A):

$$\varphi_{on-fiber}(r,\omega,F,z_{prop}) = \tilde{\varphi}(r,\omega,F) + \varphi_{div}(r,\omega,z_{prop}). \tag{21}$$

The corresponded group delay of the achromatic metalens on fiber is (Fig. S5A):

$$\frac{d\tilde{\varphi}_{on-fiber}}{d\omega} = \frac{d\tilde{\varphi}}{d\omega} + \frac{d\varphi_{div}}{d\omega}. \tag{22}$$

Supplementary Note 5. Time-bandwidth product of a waveguide-type achromatic metalens

The fundamental limit for working frequencies in an achromatic metalens can be understood with the aid of the TBP which is a product of the temporal duration ΔT and the spectral bandwidth $\Delta\omega$. The TBP of a designed achromatic lens with the desired time delay and bandwidth $(\Delta T\Delta\omega)_{Lens}$ must be smaller than the TBP of the constructed meta-atom library $(\Delta T\Delta\omega)_{Library}$ and the upper bound TBP κ:

$$(\Delta T\Delta\omega)_{Lens} \leq (\Delta T\Delta\omega)_{Library} \leq \kappa. \tag{23}$$

The required time delay of achromatic lens is[4]:

$$\Delta T = \frac{f}{c}\frac{1}{\Theta\left(\frac{NA}{n_m}\right)}, \text{ where } \Theta\left(\frac{NA}{n_m}\right) = \frac{\sqrt{1-\left(\frac{NA}{n_m}\right)^2}}{1-\sqrt{1-\left(\frac{NA}{n_m}\right)^2}}, \tag{24}$$

where c is the speed of light, NA is the numerical aperture of the achromatic lens, and

n_m is the refractive index in the focal region. The achievable bandwidth $\Delta\omega$ of the achromatic lens using the meta-atom library is calculated as:

$$\Delta\omega \leq \frac{(\Delta T\Delta\omega)_{Library}}{\Delta T} = (\Delta T\Delta\omega)_{Library}\frac{c}{f}\Theta\left(\frac{NA}{n_m}\right). \quad (25)$$

TBP based on library data is considered as the maximum value of modified group delay in the meta-atom library, with the condition that both the phase and modified group delay responses up to the maximal values are fully covered (Fig. 2D). By using the above equations, the TBP of our library data was calculated (Table 1), and plotted as solid lines in Fig. S5B-S5C.

Supplementary Note 6. Polarization-insensitive 3D meta-atom design based on cross polarization.

According to the Jones matrix, the electric field passing through a single subwavelength birefringent nanopillar waveguide can be defined as:

$$\begin{bmatrix}\tilde{E}_x\\ \tilde{E}_y\end{bmatrix} = \frac{\tilde{t}_l+\tilde{t}_s}{2}\begin{bmatrix}1\\ \pm i\end{bmatrix} + \frac{\tilde{t}_l-\tilde{t}_s}{2}e^{\pm i2\alpha}\begin{bmatrix}1\\ \mp i\end{bmatrix}, \quad (26)$$

where $\pm$ and $\mp$ represent the co- and cross-polarization responses, respectively; $\tilde{E}_{x,y}$: output electric field along the Cartesian coordinates *x* and *y*-axis; $\tilde{t}_{l,s}$: complex-amplitude transmission coefficients of the meta-atom along short and long axis; *α*: in-plane rotation angle of a meta-atom. For the meta-atoms with asymmetric in-plane geometry, it can convert input circular polarization into the opposite, for instance, left-handed circular polarization to right-handed circular polarization or vice versa. The cross-polarization term in Eq. 26 consists of a geometric phase (polarization-dependent) term $e^{\pm i2\alpha}$ that depends on the in-plane rotation angle of the meta-atom,

as well as a polarization-independent term $\frac{\tilde{t}_l-\tilde{t}_s}{2}$ that contributes to the propagation phase.

Notably, when we set the rotation angle α to be constant (0 and 90 degrees in our case) in Eq. 26, the whole cross-polarization term becomes polarization independent, leading to polarization-insensitive phase and group delay (GD) responses. In this case, both left- and right-handed circular polarization components can be converted into the same amplitude and phase responses. For examples, when α=0, the outputs of both the LCP and RCP input components lead to the same complex-amplitude response in the cross-polarization (see SI Table 1). Similarly, when $\alpha = \pi/2$, the outputs of the LCP and RCP input components are converted into the same cross-polarization response. As such, the polarization conversion efficiency can be calculated as $\left|\frac{\tilde{t}_l-\tilde{t}_s}{2}\right|^2$. Therefore, an arbitrary input polarization, which can be regarded as the combination of orthogonal LCP and RCP polarization components, can lead to the same cross-polarization response.

SI Table 1. Transmitted electric fields $\begin{bmatrix}\tilde{\boldsymbol{E}}_x\\ \tilde{\boldsymbol{E}}_y\end{bmatrix}$ after passing through a subwavelength birefringent meta-atom.

	For an input LCP component	For an input RCP component
**α=0**	$\frac{\tilde{t}_l+\tilde{t}_s}{2}\begin{bmatrix}1\\ i\end{bmatrix}+\frac{\tilde{t}_l-\tilde{t}_s}{2}\begin{bmatrix}1\\ -i\end{bmatrix}$	$\frac{\tilde{t}_l+\tilde{t}_s}{2}\begin{bmatrix}1\\ -i\end{bmatrix}+\frac{\tilde{t}_l-\tilde{t}_s}{2}\begin{bmatrix}1\\ i\end{bmatrix}$
**α= π/2**	$\frac{\tilde{t}_l+\tilde{t}_s}{2}\begin{bmatrix}1\\ i\end{bmatrix}-\frac{\tilde{t}_l-\tilde{t}_s}{2}\begin{bmatrix}1\\ -i\end{bmatrix}$	$\frac{\tilde{t}_l+\tilde{t}_s}{2}\begin{bmatrix}1\\ -i\end{bmatrix}-\frac{\tilde{t}_l-\tilde{t}_s}{2}\begin{bmatrix}1\\ i\end{bmatrix}$

The GD of meta-atom can be understood as the first-order derivative of the phase response of the meta-atom with respect to frequency, namely $\frac{\partial}{\partial\omega}\left(\frac{\tilde{t}_l-\tilde{t}_s}{2}\right)$. The calculated GD values of our meta-atoms span from 50 to 72 fs (Fig. S6). Accordingly, the modified GD of our meta-atom is defined as

$$\text{Modified } GD = GD - min(GD), \tag{27}$$

where the minimum of GD in our case is set to be 58 fs. Therefore, the modified GD of our library spans from -8 to 14 fs (Fig. 2C and D).

Supplementary Note 7. Meta-atom size and pitch considerations.

Here we kept a constant pitch distance for all meta-atoms throughout the metalens surface. The geometry of meta-atom was optimized to maximize its interaction with incident light. In general, the effective length (the product of refractive index and geometric length) of meta-atom should be comparable to the incident wavelength. Given the low refractive index of our used polymer meta-atoms, the required geometric length can be approximated as 1.1 μm ($\frac{1.65\text{um } (nominal\ wavelength)}{1.5\ (refractive\ index)}$). Given the fabrication constraints of two-photon laser lithography, we considered a duty cycle of 50% in a meta-atom unit cell, and therefore we used a pitch distance of 2.2 μm in our paper. It should be mentioned that under this pitch distance, meta-atoms with different in-plane aspect ratios exhibit high cross-polarization conversion efficiency (SI Fig. 1), which is crucial for designing an achromatic metalens of high efficiency.

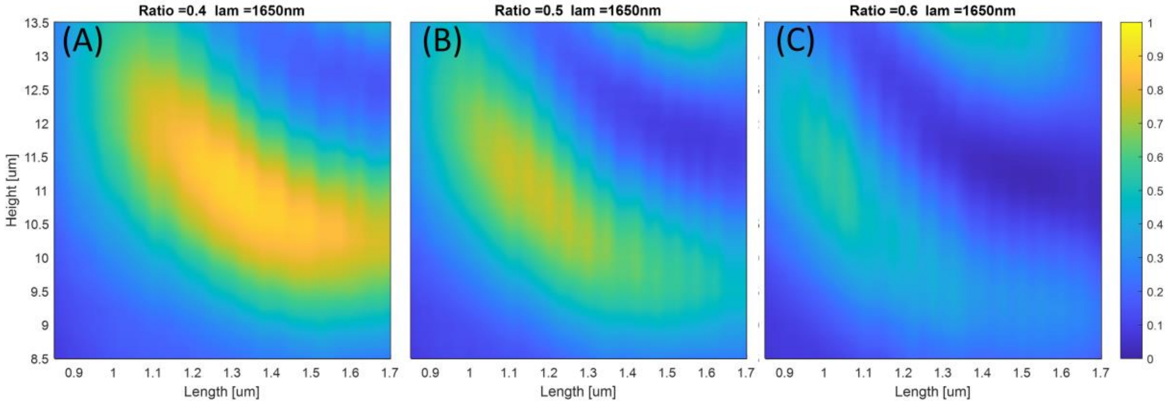

SI Fig. 1. Polarization conversion efficiency of 3D meta-atoms with fixed in-plane aspect ratios of 0.4 (A), 0.5 (B) and 0.6 (C) at a nominal wavelength of 1.65 μm. The pitch distance of meta-atoms was fixed at 2.2 μm.

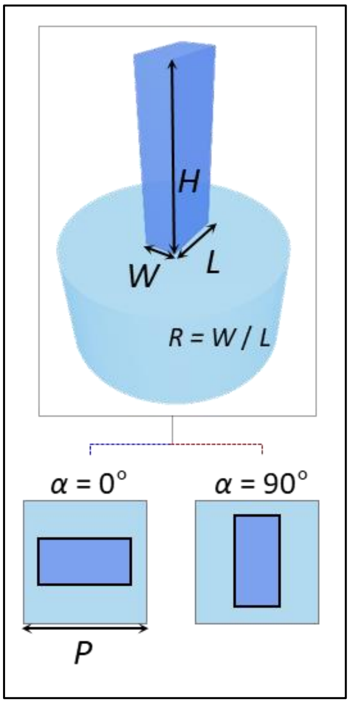

Second, the near-field coupling between adjacent meta-atoms should also be considered. To clarify this point, we simulated effective refractive indices of the transverse electric (TE) and transverse magnetic (TM) modes of the 2D cross-sections of 3D nanopillars (inset figure) across the wavelength of interests, wherein the pitch of nanopillar meta-atom was varied from 1.7 μm to 10 μm (SI Fig. 2). We simulated the effective refractive indices of a meta-atom with a length of 1.625 μm and a width of 0.813 μm. In particular, the effective refractive indices are sharply increased when the pitch gets smaller than 2 μm, indicating modal hybridization and thus a strong near-field coupling between adjacent meta-atoms. On the other hand, meta-atoms with a pitch of above 3 μm show a nearly constant effective refractive index and thereby a negligible near-field coupling between adjacent meta-atoms.

However, the increase of pitch will reduce the metalens efficiency, due to the Bragg diffraction in higher diffraction orders and insufficient sampling of a lens profile.

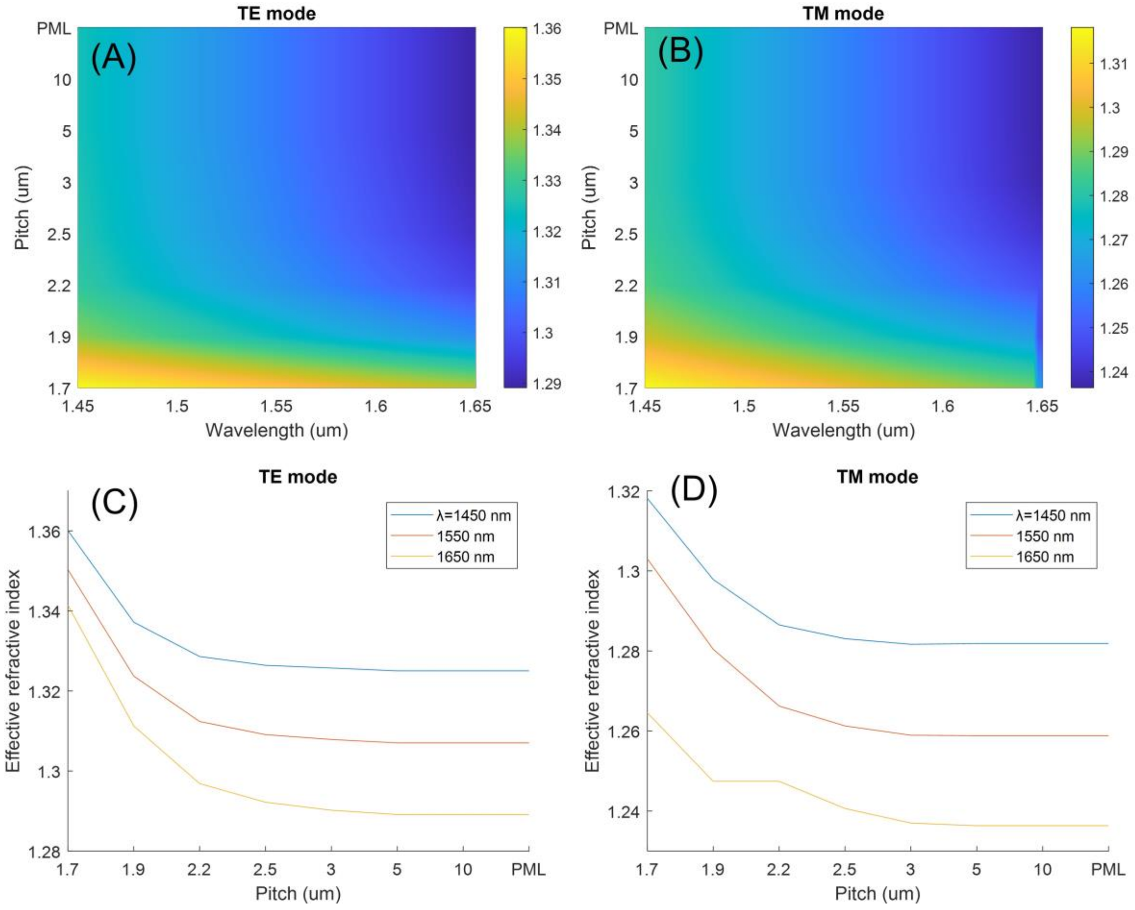

SI Fig. 2. Effective refractive indices of TE and TM modes of a 3D nanopillar across the wavelength of interest. The pitch distance was varied from 1.7 μm to 10 μm. The nanopillar transverse dimensions of length and width were fixed as 1.625 μm and 0.813 μm, respectively.

Third, we considered the relationship between the spatial sampling and focal position/focusing efficiency. Indeed, insufficient sampling causes incorrect digitalization of a lens phase function, and therefore, creating some side effects such as a focal shift and decreased focusing efficiency. For a better comparison, we further

simulated the performance of a same metalens profile under different sampling conditions, using diffraction theory based on the Fraunhofer approximation (given the fact that the NA of our metalens is small). Different sampling rates (corresponding to the metalens pitch distances) were chosen to investigate their influence on the lens performance, including focal length and focusing efficiency (SI Table 2). The focusing efficiency is defined as the integrated intensity over three times of the full width at half maximum (FWHM) area in the focal plane with respect to the integrated intensity over the whole lens aperture.

Our results indicate that a large pitch used for digitizing the lens function (with a NA of 0.2 at the wavelength of 1.65 μm, a lens diameter and a focal length of 100 μm and 240 μm, respectively) can reduce the lensing performance by shifting the focal length and reducing the lens focusing efficiency, which becomes obvious when the pitch distance is larger than 1 μm. We set a constant pitch distance of 2.2 μm in our work through balancing 1) high cross-polarization conversion efficiency; 2) sufficient sampling of a lens function with a relatively high efficiency; 3) negligible near-field coupling. Fabrication of an achromatic metalens with a pitch below 1 μm would be very challenging due to the large aspect ratio between the height and transverse dimensions, which reaches up to 33.75 in our work. Moreover, according to our simulation in SI Table 2, the pitch smaller than 1 μm can incur pronounced near-field coupling between nanopillars.

SI Table 2. Simulated focal length and focusing efficiency of a metalens function by varying the pitch distance and pixel number of the lens with a fixed numerical aperture of 0.2 (a diameter of 100 μm and a desired focal position of 240 μm).

This is equivalent to our metalens on glass designed in Fig. 2. The wavelength was fixed as 1650 nm. The used pitch distance by this work is in bold.

Pitch distance (μm)	Focal length (μm)	Focusing efficiency
4	218	0.505
3	223	0.529
2.2	229	0.607
2	231	0.615
1	236	0.747
0.5	238	0.824
0.2	239	0.886
0.1	240	0.920

Supplementary Note 8. Characterization of off-axis focusing of an achromatic metalens.

To verify the achromatic metalens performance for off-axis imaging, we have conducted a 3D Finite-Difference Time-Domain (FDTD) simulation using our selected meta-atoms for implementing the desired phase and group delay responses in Figs. 2E and 2F, respectively. We characterized the focusing performance of the achromatic metalens based on different incident angles at different wavelengths (Fig. S15). Specifically, we simulated the off-axis focusing performance based on an oblique incidence angle up to ±7.5 degrees, which correspond to the lateral shifts of ±52 μm (@1650 nm) in the focal plane (Figs. S15A and S15B). We simulated the focusing performance at three wavelengths of 1250 nm, 1450 nm and 1650 nm. Our results show that our achromatic metalens can also compensate for the chromatic aberration for off-axis incident beams for the incident angle up to ±7.5 degrees. The off-axis focusing efficiency keeps flat for an oblique incidence angle up to 5 degrees (Fig. S15C). As such, our simulation results suggest that our designed achromatic metalens (without further optimization) could exhibit a good performance for off-axis imaging up to ±7.5 degrees, which opens the possibility of implementing it on a multicore fiber or a fiber bundle for the wide-field imaging with a large field-of-view of 104 μm (@1650 nm).

To achieve wide-field imaging, we can implement our achromatic metalens with high-performing on- and off-axis imaging capability on top of a multicore fiber that consists of multiple SMFs (Fig. S16). We must mention, however, there is a general technical challenge that may limit the imaging quality of the achromatic metalens that is implemented on a multicore fiber (or a fiber bundle). Since the outputs of SMFs in the multicore fiber are all faced perpendicular to the fiber surface, the collection efficiency

for off-axis imaging through the out-ring SMFs should be reduced as compared to the on-axis case (Fig. S16). The collected off-axis imaging signal by our achromatic metalens has an angle offset with respect to the nominal fiber emission/collection direction (solid lines in Fig. S16). This angle mismatch could reduce the collection (fiber coupling) efficiency for off-axis light signals and hence limit the field-of-view of an achromatic metalens, although the use of a multicore fiber could mitigate this mismatch. We believe this technical challenge is intrinsic to the use of a multicore fiber or a fiber bundle for wide-field imaging, which should also apply to Refs. 5 and 6.

Supplementary Note 9. Laser damage threshold of a polymer metalens.

To assess how the nanoprinted structures are affected by high light intensities, (A) the damage threshold of polymers based on literature values was estimated and (B) additional experiments on nanoprinted test samples were performed.

A. Comparison of the fluence in the experiments performed with the polymer damage fluence: The damage threshold of materials is within the context of ultrafast laser pulses is typically defined by the damage fluence F_d (unit: J/m^2) which for polymeric material is of the order of F_d=3500 J/m^2 (see for instance *AIP Conference Proceedings* **1278**, 56 (2010); https://doi.org/10.1063/1.3507148). The fluence F_{max} that maximally appears in the experiments presented here was estimated by dividing the maximum single-pulse energy E_{max} = 2.13nJ (calculated at highest average output power $P_{ave,max}$=170mW, τ = 30fs, ν = 80MHz, λ = 1550 nm) and the area of the metalens A_m=π·(100 μm/2)^2=7.85·10^{-9}m^2, leading to F_{max} = E_{max}/A_m= 0.27 J/m^2. Comparing this value with the damage fluence F_d, it can be seen that in the current experiment the damage threshold is undercut by a factor of 10000, thus clearly suggesting that polymer-based metasurfaces used here can be used in ultrafast nonlinear optics.

B. Power stability experiments on test sample: To quantify the power handling capabilities of the polymer metasurfaces, we performed additional experiments using a femtosecond fiber laser operating at 1.55μm (Toptica FemtoFiber pro IRS-II). As a test sample, we printed a planar film-type structure on a glass surface that had a diameter larger than the diameter of the metalens used in the main text. Through gently focusing the light from the ultrafast laser and placing the samples at an appropriate position after the focus (about 2mm, Supplementary Fig. S17A), a beam diameter of roughly 100μm was reached, matching the size of the metalens used in this work. This ensures that the intensity distributions can be quantitatively compared between these experimental runs and the metalens experiments reported in the manuscript.

Via this arrangement, the nanoprinted sample was exposed to a sequence of ultrashort laser pulses over a defined duration of time and the transmitted power was recorded every minute. The measurements show that even at the maximum average output power of the laser (Supplementary Fig. S17B), no change in output power is observed over a period of one hour, indicating that the polymer structure is able to withstand the high intensity pulses. This observation is confirmed by additional microscopic inspection of the sample after the one-hour exposure, showing no visible change or degradation of the nanoprinted part. We would like to mention that at the maximum output power of the laser, the peak power of the single pulse is more than 50 kW, a value higher than that required in many solid-state fiber-based nonlinear frequency conversion systems (see for instance *Optics Express* **21**, 10969-10977 (2013)).

C. Previous study on optical trapping: We would also like to mention that a different

type of 3D nanoprinted metalens was used in a previous study for optical trapping experiments [Ref. 7]. In the experiment reported there, cw-laser power levels of the order of 50mW in the red spectral domain have been used, showing no degradation of the nanoprinted structure for any of the experiments (which partially went over hours), thus again confirming that metalens can withstand a substantial amount of laser power.

Based on the above points, it can be clearly stated that due to the comparatively large transverse extent of the metasurface structure (on the order of 100 μm), the local light fluence in commonly used experimental configurations is sufficiently low to prevent damage to the nanoprinted structure. Both investigations confirm that the use of typical ultrafast laser pulses is unproblematic in the context of our metafiber device.

Supplementary Figures

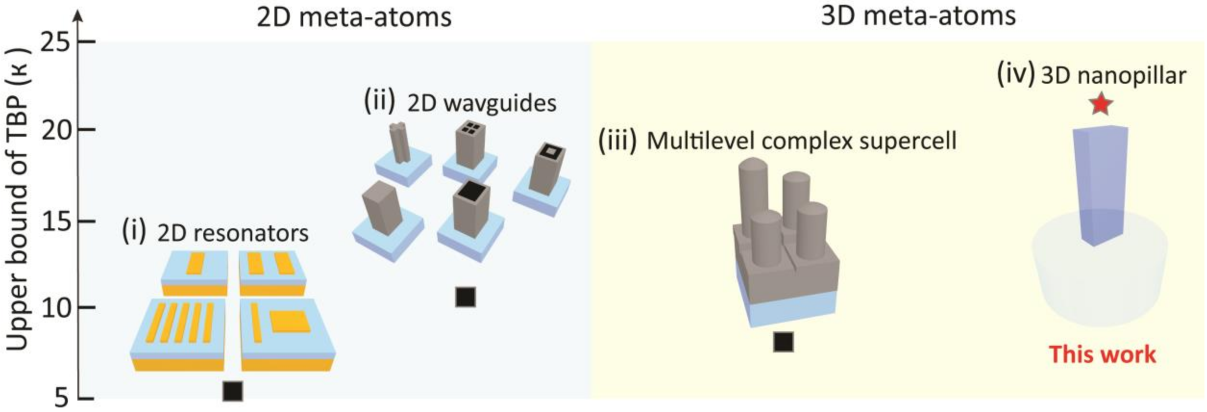

Figure S1. Performance comparison of different achromatic metalenses demonstrated in the fiber-relevant telecommunication band, in which the upper bound of the TBP of their meta-atoms are presented. Schematic diagrams of the meta-atoms in (a), (b), and (c) are adapted from References 1, 8, and 4, respectively.

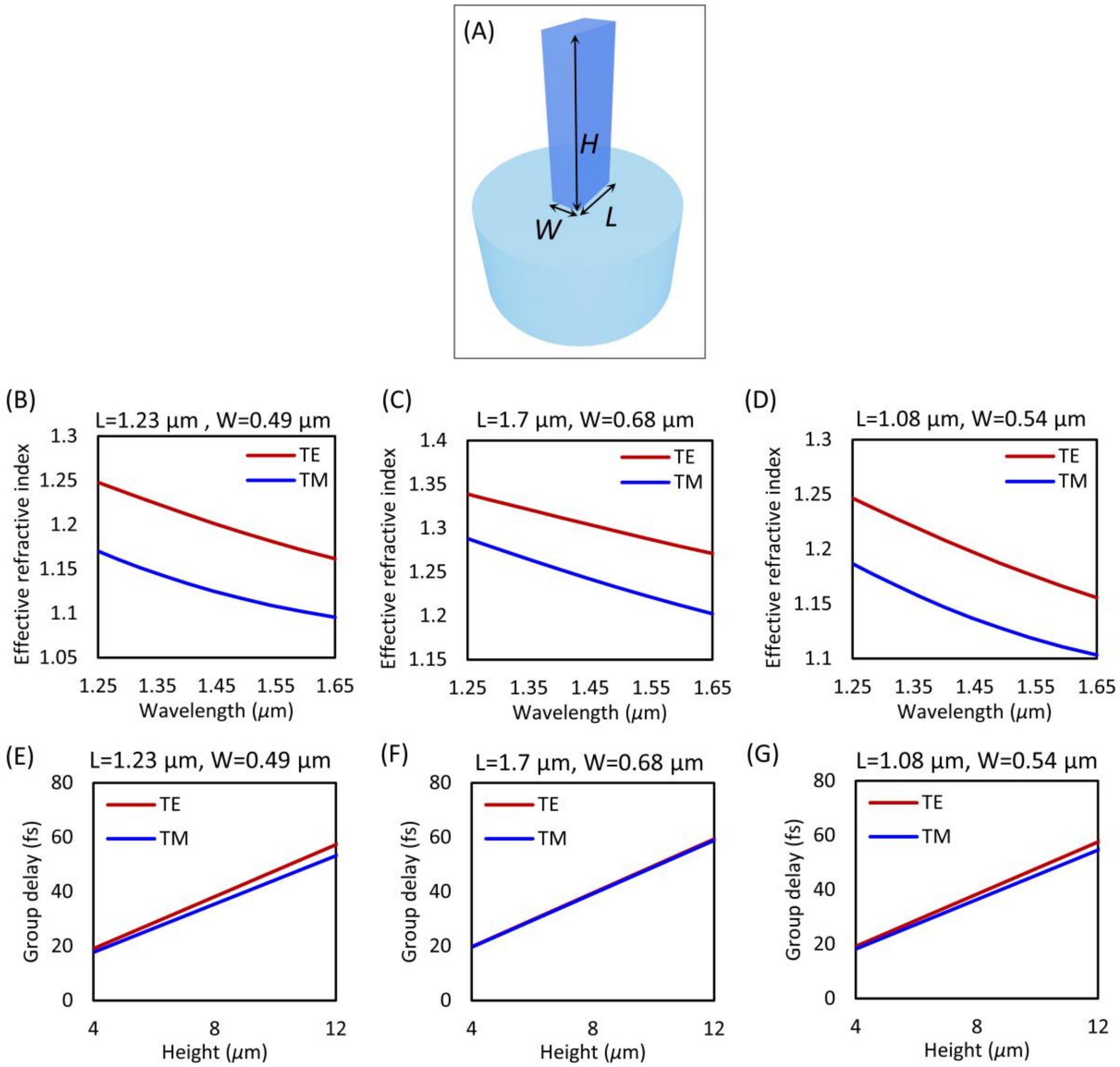

Figure S2. Effective refractive indices and group delay responses of 3D nanopillar meta-atoms. (**A**) Schematic illustration of a nanopillar waveguide, with longitudinal height *H*, transverse width *W* and length *L*, respectively. (**B**-**D**) Effective refractive indices of the transverse electric (TE) and transverse magnetic (TM) modes as a function of wavelength for nanopillar with transverse dimensions of L = 1.23 μm, W = 0.49 μm (B); L = 1.7 μm, W = 0.68 μm (C); and L = 1.08 μm, W = 0.54 μm (D), respectively. (**E**-**G**) Group delay responses of the nanopillars in (B-D).

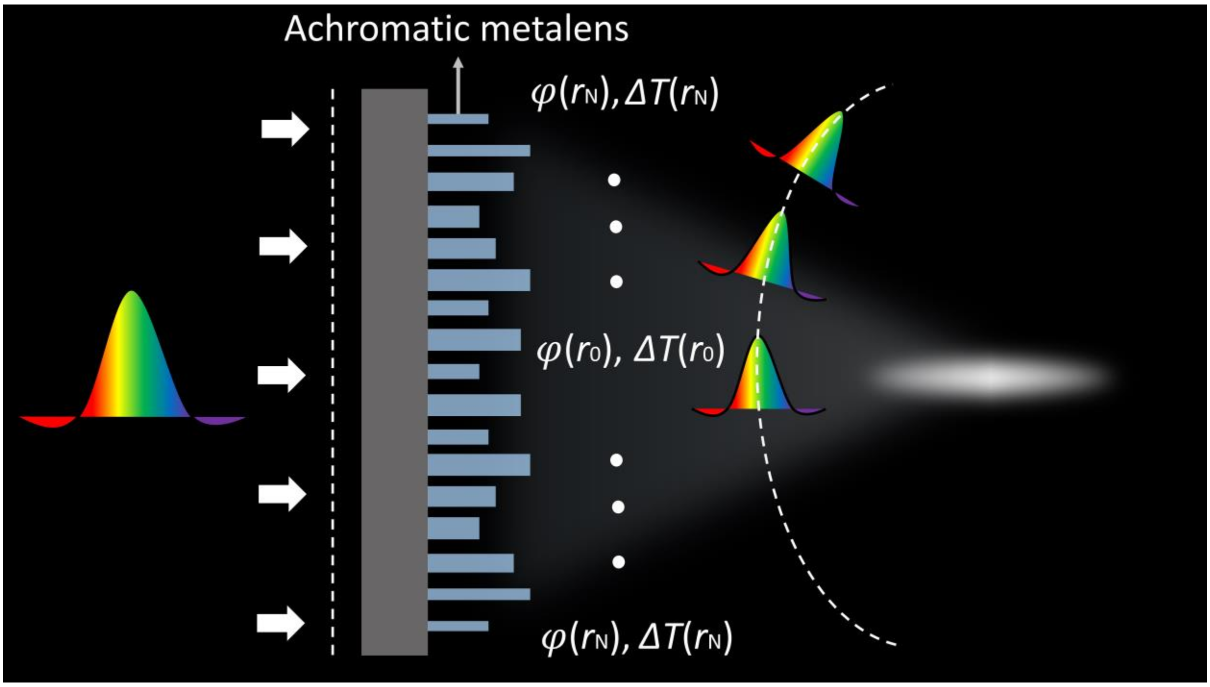

Figure S3. Principle of an ultrathin diffractive achromatic metalens. To realize achromatic focusing, the radially (*r*) arranged phase profile of $\varphi(r)$ creates a spherical wavefront for focusing, while the group delay profile of $\Delta T(r)$ compensates the difference in arrival times at the focus for broadband light.

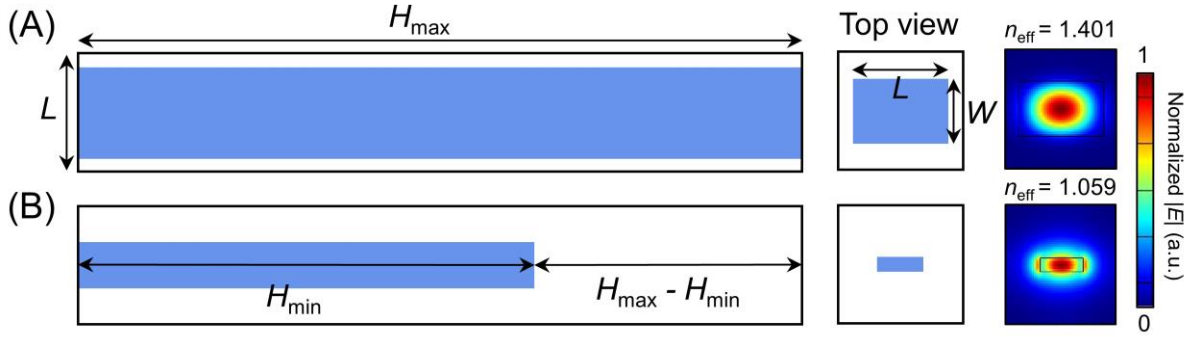

Figure S4. The maximal and minimal phase delays imposed by 3D meta-atoms. (**A**) An example of a 3D meta-atom with the maximal phase delay. H_{max}: 13.5 um; L: 1.7um; R: 0.6; the effective refractive index n_{eff}^{max} : 1.401. (**B**) An example of a 3D meta-atom with the minimal phase delay. H_{min}: 8.5 um; L: 0.85um; R: 0.3; the effective refractive index n_{eff}^{min} : 1.059.

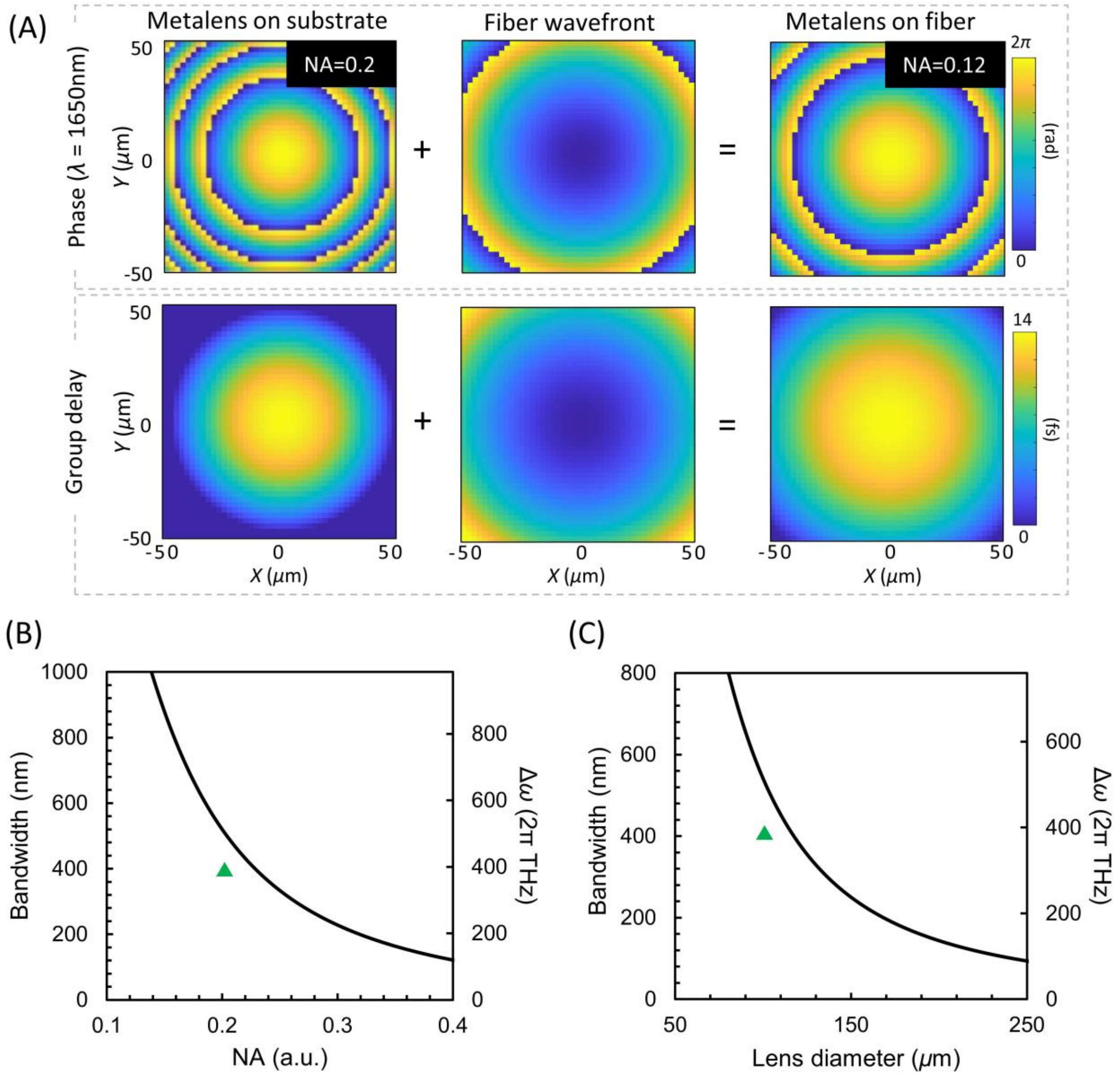

Figure S5. Theoretical design of an achromatic metalens based on the consideration of the TBP limit. (**A**) Phase and group delay profiles of a spherical lens and a SMF output beam (at a distance of 525 μm) for the design of an achromatic metalens on a substrate and on a fiber end face, respectively. The designed was made at the maximal wavelength (1650 nm) within the working bandwidth. (**B** and **C**) The TBP bandwidth limit of an achromatic metalens as functions of the NA (B) and the aperture size (C) of a metalens, respectively. The focal distance is fixed at f = 250 μm. The center wavelength was set to be 1450 nm. Black curves present the results based on our 3D meta-atom design library. Green dot: our current achromatic metalens design in this paper.

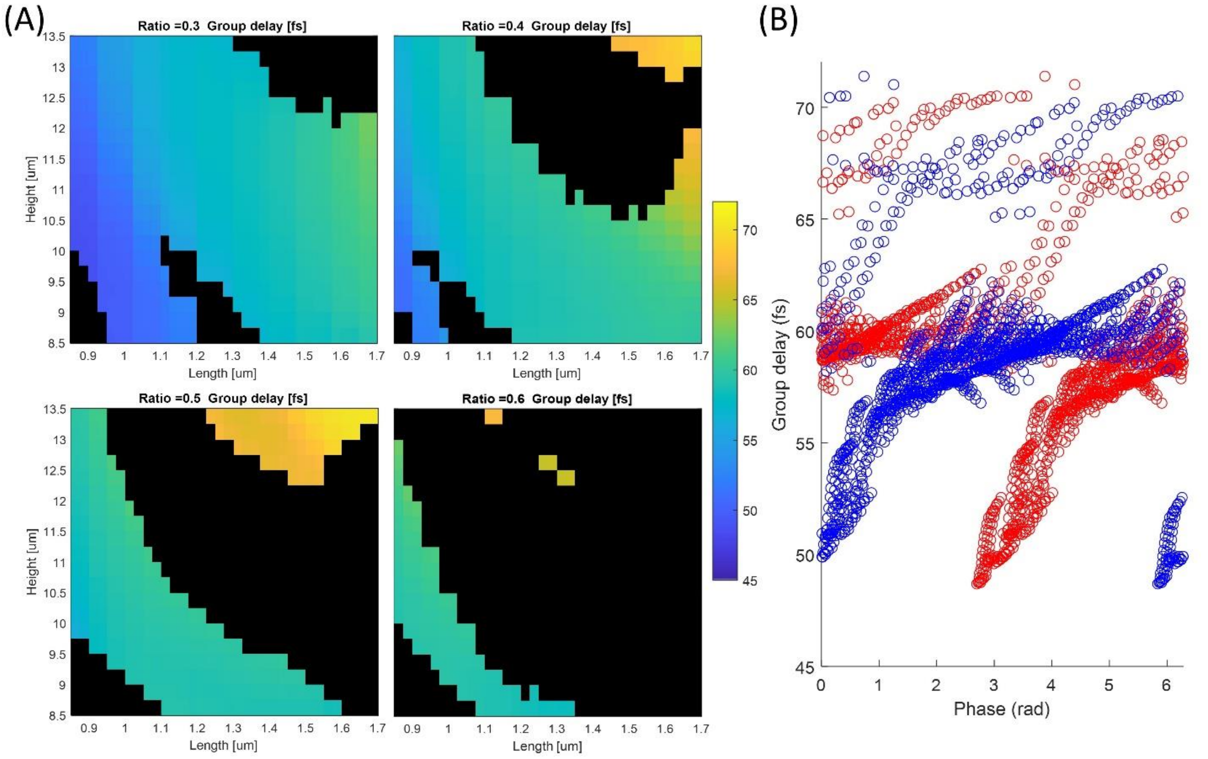

Figure S6. 2D colormap of relative group delay of 3D IP-L nanopillar waveguides. *R*: in-plane ratio between length and width. The structures which have conversion efficiencies less than 0.1 are excluded.

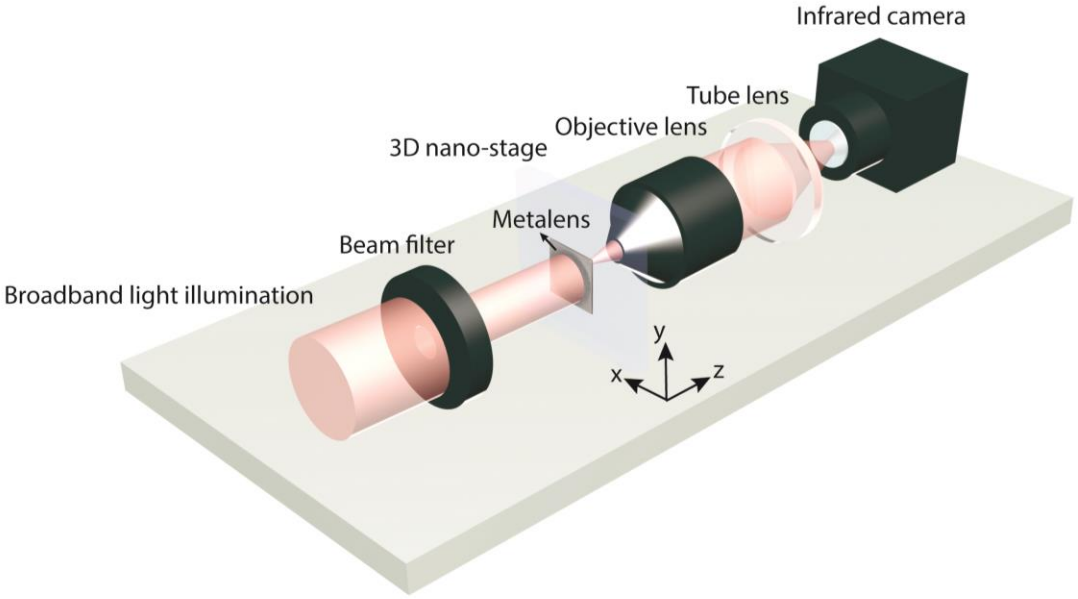

Figure S7. Optical setup for characterizing the focusing performance of a 3D-nanoprinted achromatic metalens on a glass substrate.

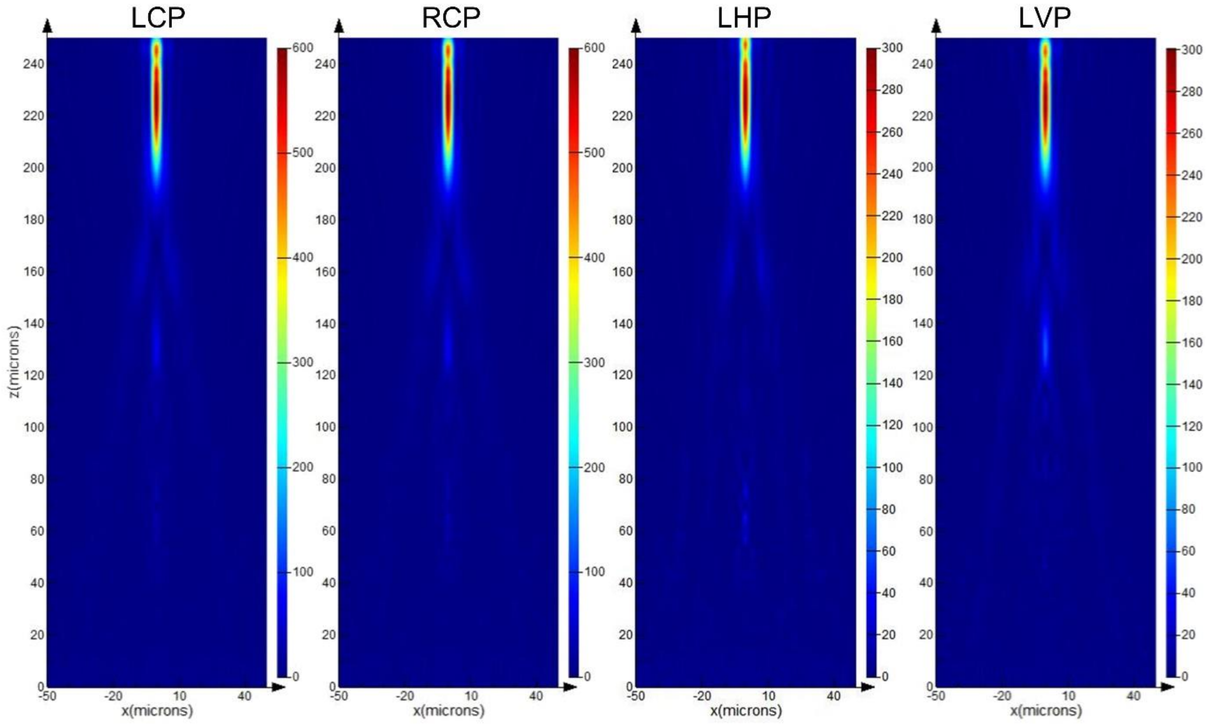

Figure S8. Polarization-insensitive focusing of the 3D achromatic metalens. The axial focal distribution of our designed 3D achromatic metalens is simulated at a wavelength of 1650 nm using finite-difference-time-domain method. The incident polarization state was set to be left- and right-handed circular polarization (LCP and RCP), and linear polarization along horizontal and vertical directions, respectively.

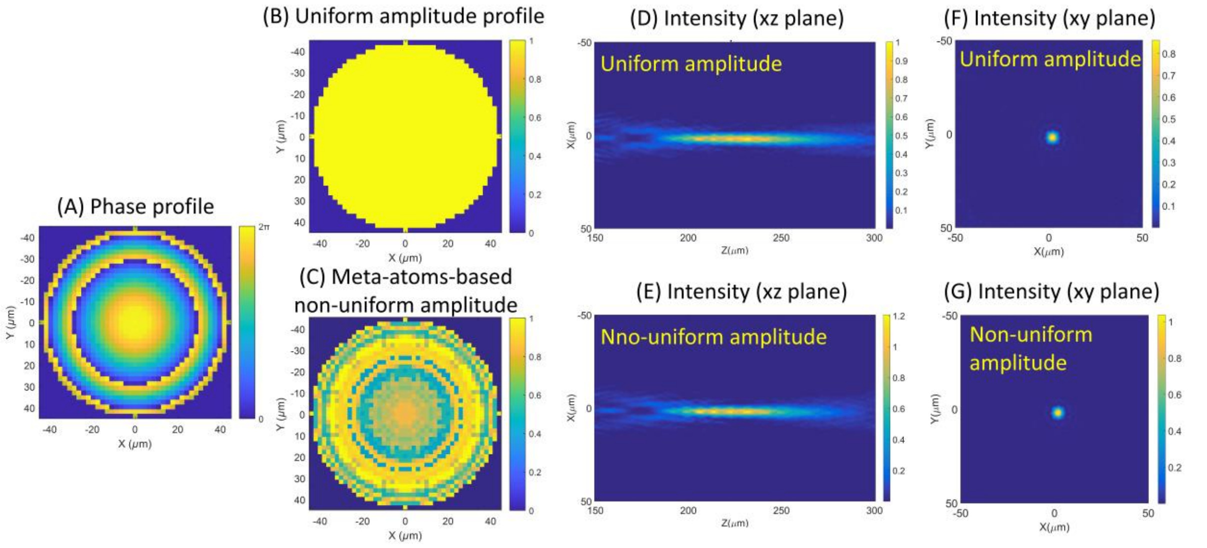

	Focal length (μm)	Focusing efficiency	FWHM (μm)
Uniform amplitude	228	0.607	4.604
Non-uniform amplitude	224	0.657	4.604

Figure S9. Comparison of the lens performance between a uniform amplitude profile and a non-uniform amplitude profile extracted from metalens design in Figure 2 in the main text. (**A**) The phase profile of a spherical lens function (used by our metalens). (**B**) A uniform amplitude profile. (**C**) Meta-atoms-based non-uniform amplitude profile. (**D** and **E**) Intensity distributions of the lens function in the XZ focal plane with a uniform amplitude profile (D) and with a non-uniform amplitude profile (E). (**F** and **G**) The counterparts of (D and E) showing the intensity distributions in the transverse XY focal plane. The table compares focusing performance of lenses with uniform and non-uniform amplitude profiles.

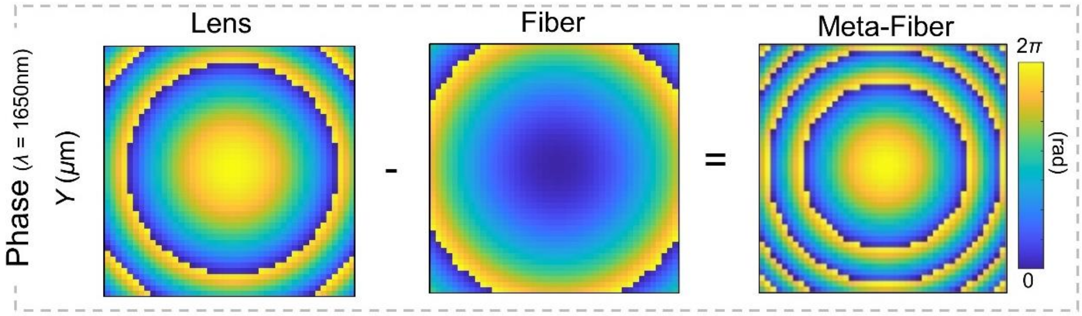

Figure S10. Design of a chromatic metalens. 2D phase maps of the chromatic lens, fiber, and modified lens. Group delay in all pixels is fixed to 4 fs for comparison (no group delay compensation).

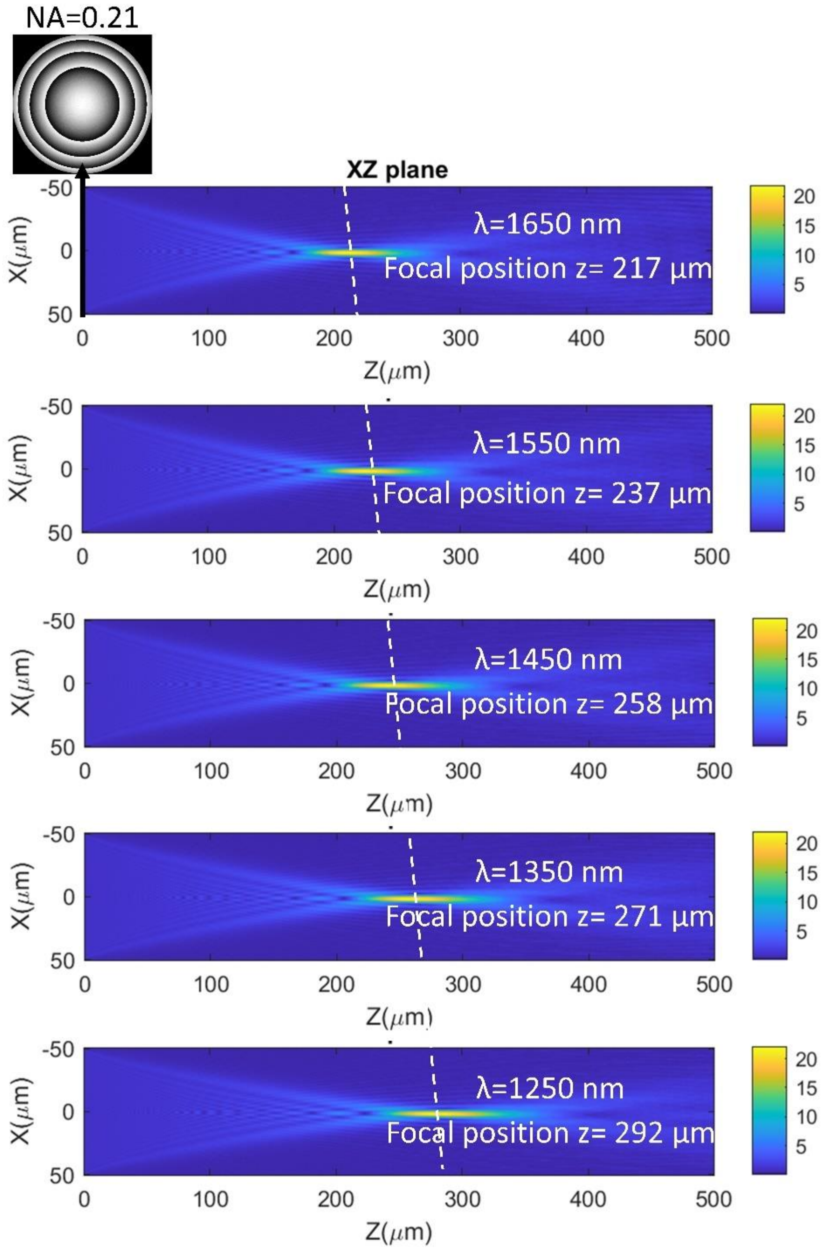

Fig. S11. Theoretical analysis of the axial focal shift of using a spherical lens profile designed at a single wavelength of 1.65 μm, without compensating the chromatic aberration.

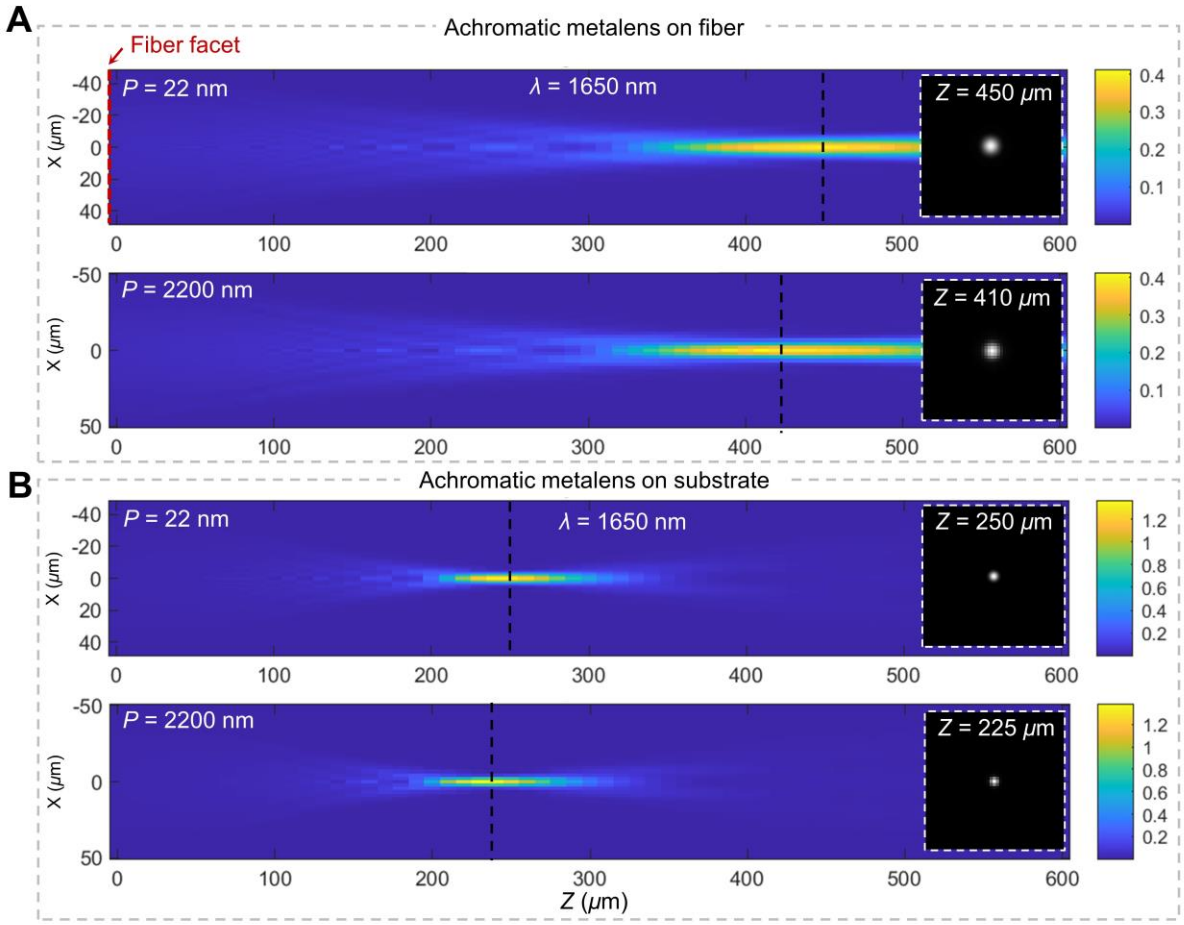

Figure S12. Simulated foci of achromatic metalens on fiber and glass substrate, respectively. (**A**) Achromatic metafiber focus with pixel pitch of *P* = 22 (top) and 2200 nm (bottom), respectively. (**B**) The focus of the achromatic metalens on glass with pixel pitch *P* = 22 (top) and 2200 nm (bottom), respectively.

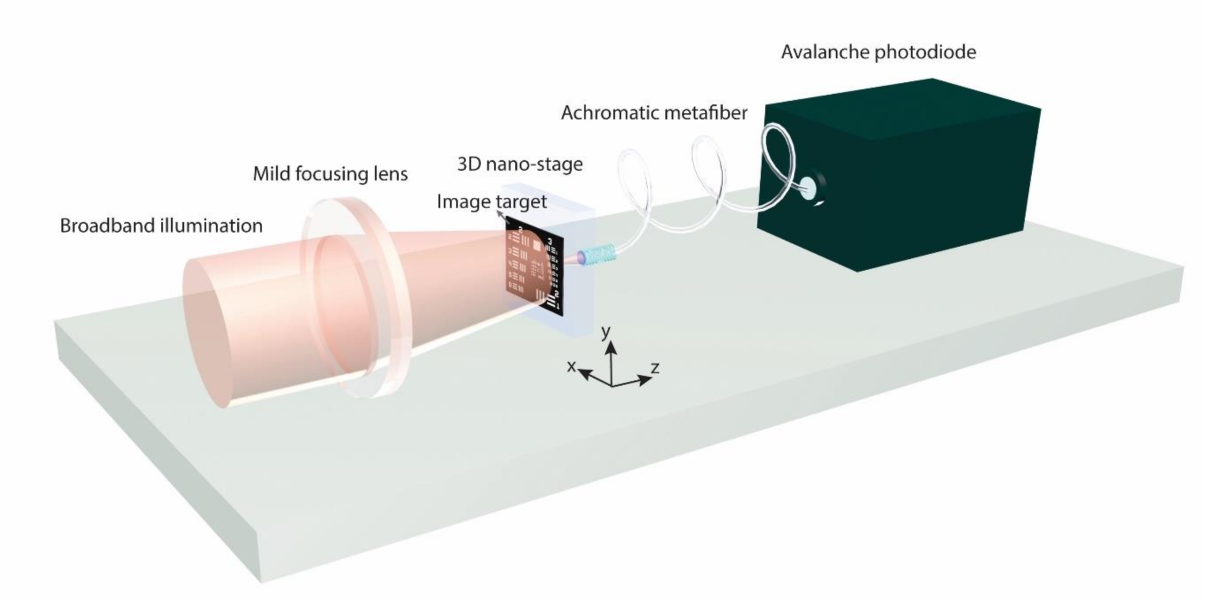

Figure S13. Optical setup for fiber-optic confocal imaging using our 3D-nanoprinted achromatic metafiber, without involving any other imaging components.

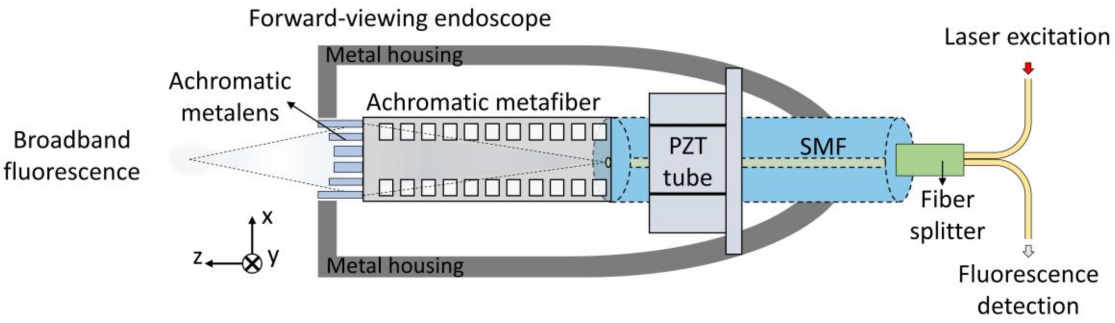

Figure S14. Suggestion for an achromatic metafiber on a forward-viewing endoscope for practical endoscopic imaging. A fiber-based beamsplitter is used to separate fluorescence signal from the laser excitation. A forward-viewing endoscope integrates an achromatic metafiber with a piezoelectric transducer (PZT) tube for distal-end confocal scanning imaging in the focal region. The achromatic metafiber can simultaneously excite and collect broadband fluorescence signals for practical endoscopic imaging.

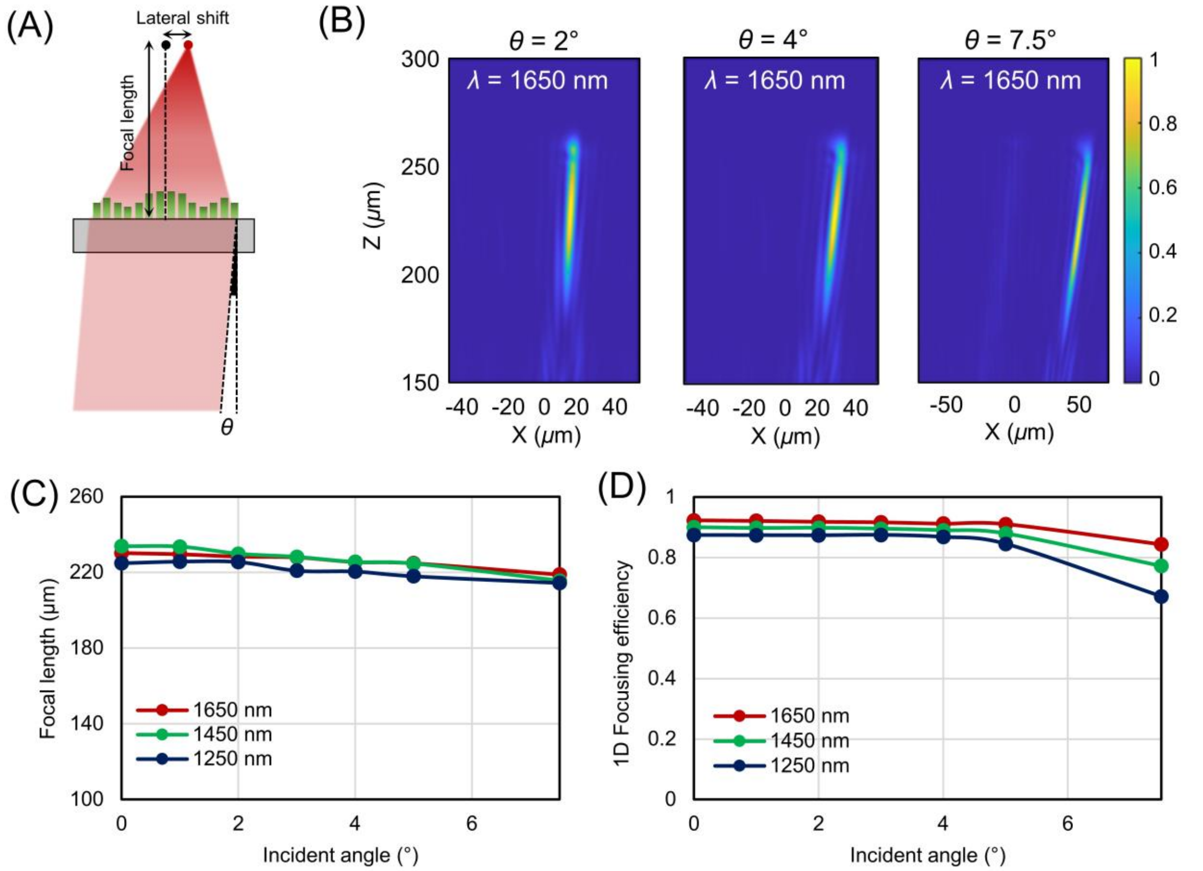

Figure S15. Numerical characterization of off-axis focusing of an achromatic metalens. (**A**) Schematic of a 3D FDTD model used for studying the performance of off-axis focusing of an achromatic metalens on a glass substrate. (**B**) Intensity distributions of the metalens focus in the longitudinal focal plane based on the incident angles of 2°, 4° and 7.5° at a wavelength of 1650 nm. Characterization of (**C**) the focal length and (**D**) focusing efficiency of the achromatic metalens under different incident angles at three wavelengths of 1250 nm, 1450 nm and 1650 nm.

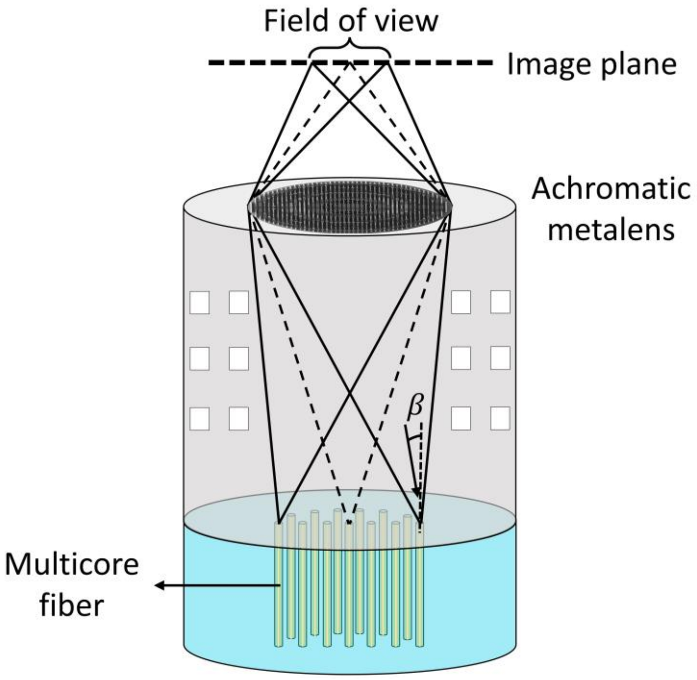

Figure S16. Schematic of an implementation of an achromatic metalens on top of a multicore fiber for wide-field imaging. The light paths of on- (dashed lines) and off-axis (solid lines) imaging are shown. The off-axis light paths induce an angle offset β with respect to the nominal emission/collection direction of the SMF, which can reduce the collection efficiency of off-axis light signals and limit the field-of-view of the metalens.

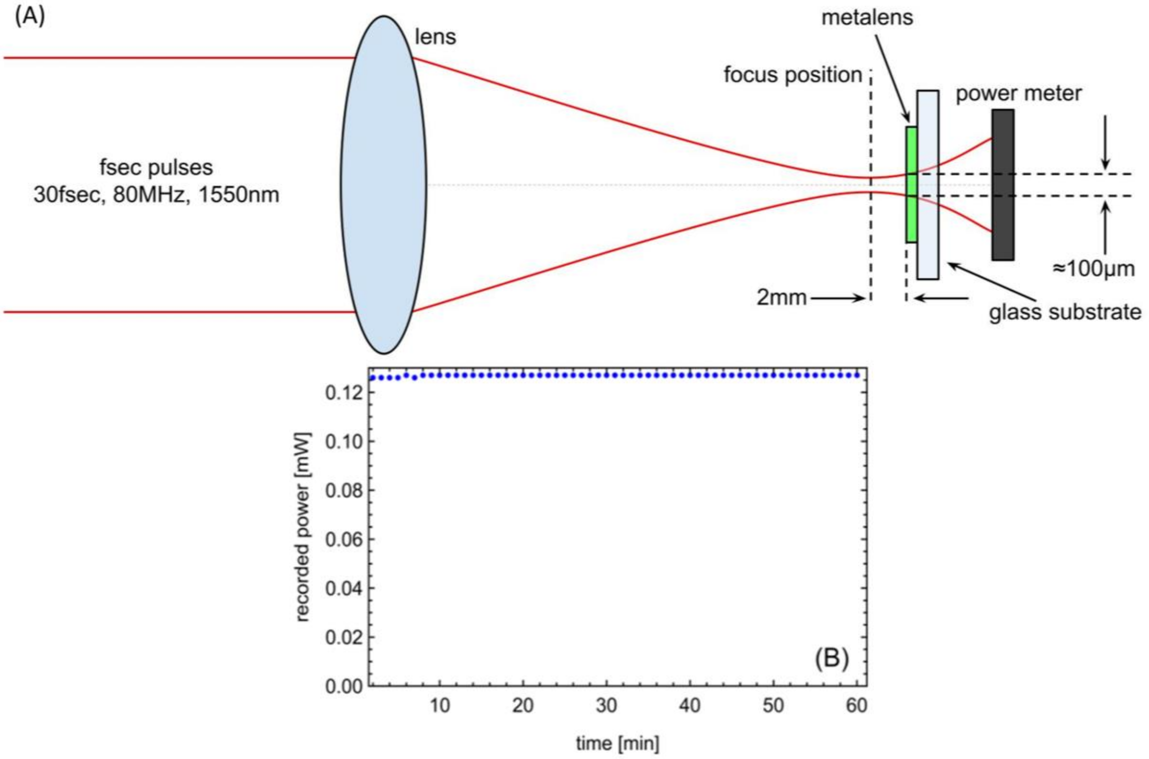

Figure S17. Laser damage threshold of a polymer metalens. (**A**). Experimental setup to investigate the damage threshold of nanoprinted structures. This arrangement was chosen to achieve similar beam expansion to the fiber-based experiments. (**B**). Recorded power at selected points of time at maximum laser power.

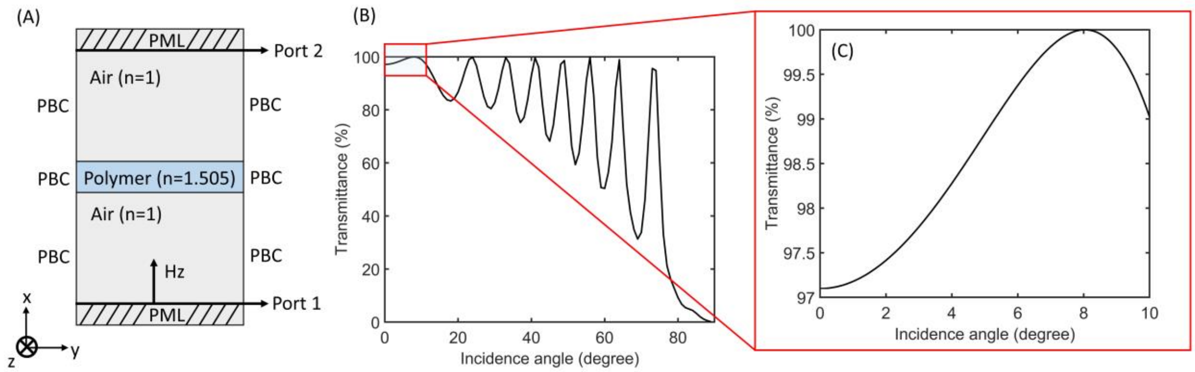

Figure S18. Transmission efficiency of a polymer slab with a thickness of 15 μm based on the illumination of light (wavelength: 1.45 μm) at different incidence angles. (**A**) Schematic of the considered numerical model. PBC: periodic boundary condition, PML: perfect matched layer. (**B**) Transmittance as a function of incidence angle from 0 to 89 degrees. (**C**) Transmittance based on the enlarged incidence angle range (up to 10 degrees) of which our achromatic metalens (NA= 0.21) is designed.

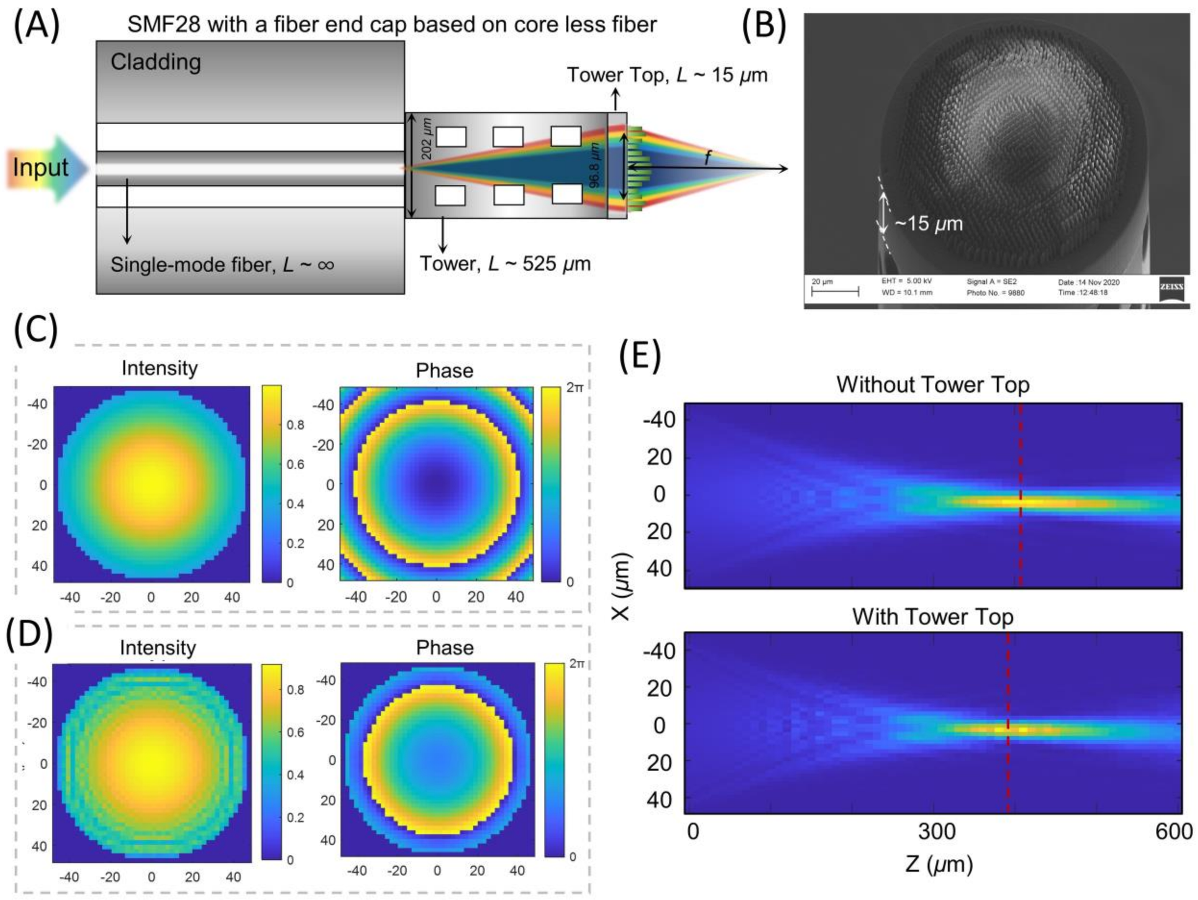

Figure S19. Numerical simulation of the influence of a polymer spacer layer (thickness of 15 μm) on the focusing performance of the achromatic metafiber. (**A**) Schematic of a 3D-nanoprinted achromatic metalens interfaced with a SMF-28, wherein a polymer-based spacer layer with a thickness of 15 μm is printed on top of the hollow tower structure to smooth the tower top. (**B**) SEM image of the printed metafiber device, in which the spacer layer is labelled out. (C and D) Simulated intensity and phase profiles of the fiber outputs without (C) and with (D) the spacer layer on tower top, respectively. (**E**) Simulation results of the focusing performance based on the beam profiles in (C) and (D).

Table 1. Summary of key features of achromatic metalenses demonstrated in the fiber-optic communication wavelength band.

Refs	NA	D/μm	Δλ/μm	$\Delta T \Delta\omega$ (Lens)	$\Delta T \Delta\omega$ (Library)	κ	**Focusing efficiency (%)**	Notes
This paper	0.2	100	1.25−1.65	6.01	8.29	21.34	24	▪ Polarization-insensitive
Ref. 33	0.24	100	1.2−1.65	8.74	3.14	11.50	60	▪ Polarization-insensitive
	0.13	200	1.2−1.65	9.24	3.14	11.50	-	
Ref. 34	0.22	55.55	1.2−1.65	4.36	5.76	-	50	▪ Polarization-sensitive
	0.27	55.55	1.2−1.65	5.47	5.76	-	35	
	0.32	55.55	1.2−1.65	6.75	5.76	-	-	
Ref. 35	0.27	20	1−1.8	3.36	-	7.21	7	▪Polarization-insensitive
	0.34	20	1−1.8	4.35	-	10.78	7	

Table 2. Key parameters of 3D meta-atoms used for the design of an achromatic metalens.

r **(*μ*m)**	***H*** **(*μ*m)**	***L*** **(*μ*m)**	**Aspect Ratio _*R***	**Rotation angle_*α* (°)**	**Phase (rad)**	**Modified GD (fs)**
0	13.5	1.625	0.5	0	6.145191	12.30935214
2.2	13.5	1.625	0.5	0	6.145191	12.30935214
4.4	13.5	1.625	0.5	0	6.145191	12.30935214
6.6	13.5	1.6	0.5	0	6.038003	12.28001077
8.8	13.5	1.575	0.5	0	5.581998	12.21445306
11	13.5	1.55	0.5	0	5.46474	12.17449962
13.2	13.5	1.525	0.5	0	4.990234	11.77464104
15.4	13.25	1.5	0.5	0	4.726895	11.25748274
17.6	13.25	1.65	0.4	0	4.005997	11.50103522
19.8	13.25	1.625	0.4	0	3.421469	10.77848805
22	13.25	1.575	0.4	0	2.676755	10.12734391
24.2	11.75	1.65	0.4	0	1.882477	9.09902245
26.4	13.25	1.25	0.5	0	1.117675	8.692195818
28.6	13	1.375	0.5	90	3.284484	8.182008783
30.8	13.5	1.3	0.5	90	2.311152	7.908358375
33	10.5	1.7	0.4	90	1.176484	6.531354459
35.2	10.5	1.575	0.4	90	0.111098	4.701487148
37.4	11	1.7	0.3	90	5.392402	3.785466871
39.6	11	1.25	0.4	90	4.074517	2.055415499
41.8	10.5	0.9	0.6	90	2.799695	1.737175927
44	8.5	1.1	0.6	0	4.494777	0.75788425

Table 3. Transmission, focusing, and total efficiency of the 3D-nanoprinted achromatic metalens (on glass, NA=0.2). Total efficiency = Transmission efficiency × Focusing efficiency.

Wavelength (nm)	Transmission efficiency (%)	Focusing efficiency (%)	Total efficiency (%)
1250	40.6	34.8	14.13
1350	46.7	38.7	18.11
1450	56.9	43.2	24.58
1550	58.8	52.5	30.87
1650	59.5	53.8	32.01

Note: To characterize the transmission efficiency of the fabricated metalens, we considered the ratio of total electric field intensity transmitted from the metalens to the total electric field intensity of incident light. To characterize the focusing efficiency of the designed metalens, we define the focusing efficiency as the ratio of total electric field intensity in a circular aperture with three times the FWHM of the focal spot to the total electric field intensity of the transmitted light.

Table 4. Full width at half maximum of simulated and experimentally measured achromatic metalens (on glass, NA=0.2) focus at different wavelengths.

Wavelength (nm)	FWHM (μm) (Simulation)	FWHM (μm) (Experiment)
1250	3.05	3.03
1350	3.52	3.83
1450	3.82	3.79
1550	4.01	3.84
1650	4.32	4.24

Table 5. Transmission, focusing, and total efficiency of the 3D-nanoprinted chromatic metalens (on glass, NA=0.2). Total efficiency = Transmission efficiency × Focusing efficiency.

Wavelength (nm)	Transmission efficiency (%)	Focusing efficiency (%)	Total efficiency (%)
1250	56.8	35.4	20.11
1350	66.6	36.9	24.58
1450	72	37.5	27
1550	72.7	41.4	30.1
1650	73.2	42.3	30.96

Note: To characterize the transmission efficiency of the fabricated metalens, we considered the ratio of total electric field intensity transmitted from the metalens to the total electric field intensity of incident light. To characterize the focusing efficiency of the designed metalens, we define the focusing efficiency as the ratio of total electric field intensity in a circular aperture with three times the FWHM of focal spot to the total electric field intensity of transmitted light.

Table 6. Full width at half maximum of simulated and experimentally measured achromatic metalens (on fiber, NA=0.12) focus at different wavelengths.

Wavelength (nm)	FWHM (μm) (Simulation)	FWHM (μm) (Experiment)
1250	5.41	4.97
1350	5.83	5.45
1450	6.23	5.81
1550	6.63	6.27
1650	7.13	6.74

Supplementary References

[1] Wang, S. et al. Broadband achromatic optical metasurface device. *Nat. Commun*. **8**, 187 (2017).

[2] Verdeyen, J. T. *Laser Electronics*. (Prentice-Hall, 1995).

[3] Yamada, K., Watanabe, W., Li, Y., Itoh, K. & Nishii, J. Multilevel phase-type diffractive lenses in silica glass induced by filamentation of femtosecond laser pulses. *Opt. Lett.* **29**, 1846 (2004).

[4] Balli, F., Sultan, M., Lami, S. K. & Hastings, J. T. A hybrid achromatic metalens. *Nat. Commun.* **11**, 3892 (2020).

[5] Gissibl, T., Thiele, S., Herkommer, A. & Giessen, H. Two-photon direct laser writing of ultracompact multi-lens objectives. *Nat. Photonics* **10**, 554-560 (2016).

[6] Gissibl, T., Thiele, S., Herkommer, A. & Giessen, H. Sub-micrometre accurate free-form optics by three-dimensional printing on single-mode fibres. *Nat. Commun.* **7**, 11763 (2016).

[7] Plidschun, M. *et al.* Ultrahigh numerical aperture meta-fiber for flexible optical trapping. *Light Sci. Appl.* **10**, 57 (2021).

[8] Shrestha, S., Overvig, A. C., Lu, M., Stein, A. & Yu, N. Broadband achromatic dielectric metalenses. *Light Sci. Appl.* **7**, 85 (2018).
